# Long-term impact of necrotizing enterocolitis on somatosensory function in preterm born children

**DOI:** 10.1038/s41390-025-04348-8

**Published:** 2025-08-26

**Authors:** Judith A. ten Barge, Marijn J. Vermeulen, Claudia M. G. Keyzer-Dekker, Nienke Bouw, Cecile C. de Vos, Irwin K. M. Reiss, Sinno H. P. Simons, Gerbrich E. van den Bosch

**Affiliations:** 1 Department of Neonatal and Pediatric Intensive Care, Division of Neonatology, Erasmus MC - Sophia Children’s Hospital, Rotterdam, The Netherlands; 2 Department of Pediatric Surgery, Erasmus MC - Sophia Children’s Hospital, Rotterdam, The Netherlands; 3 Department of Child and Adolescent Psychiatry/Psychology, Erasmus MC - Sophia Children’s Hospital, Rotterdam, The Netherlands; 4 Center for Pain Medicine, Erasmus University Medical Center, Rotterdam, The Netherlands

## Abstract

**Background:**

Necrotizing enterocolitis (NEC) is a painful intestinal disease in preterm infants that causes severe prolonged pain requiring high amounts of analgesics. In a critical stage of neurodevelopment, pain and analgesics potentially impact sensory development. This study compared somatosensory function and sensory processing in preterm-born children with and without a history of NEC, as well as key self-/parent-reported outcomes.

**Methods:**

In this cross-sectional cohort study, preterm-born children (6–15 years old) with NEC were matched with controls. Each child underwent quantitative sensory testing in a mobile research lab at home. Parents completed questionnaires on their child’s sensory processing, behavior, executive function, chronic pain, and (health-related) quality of life.

**Results:**

Sixty-six children (22 NEC, 44 control) participated. Children with a history of NEC exhibited lower cold pain thresholds (*β* = −11 °C, 95% CI [-21;−1.0], *p* = 0.03) compared to those without NEC. Moreover, they exhibited poorer executive function, though no longer significant after correcting for confounders. No significant differences were found in sensory processing, behavioral problems, chronic pain, and (health-related) quality of life.

**Conclusion:**

Children with a history of NEC are less sensitive to cold pain, but have otherwise similar somatosensory function and sensory processing to other preterm-born children. Most self- and parent-reported outcomes are comparable to those of other preterm-born children without NEC.

**Impact:**

- Necrotizing enterocolitis in preterm infants lastingly alters their (cold) pain sensitivity.
- Most self- and parent-reported outcomes of children with a history of NEC are similar to those of other preterm-born children.
- Sensory processing and behavioral problems are common among both preterm-born children with and without NEC history.
- Our results warrant the search for protective strategies during NICU admission and thorough follow-up after NICU discharge.

## Introduction

Necrotizing enterocolitis (NEC) is a life-threatening intestinal disease, affecting ~7% of very low birth weight (VLBW, <1500 g) infants.^1^ Infants with NEC exhibit excessive intestinal inflammation with progressive necrosis that may also cause severe systemic sepsis signs with cardiorespiratory instability.^2^ Therapeutic strategies include bowel rest and antibiotic therapy, as well as surgery in approximately two-thirds of cases.^3^ Next to cardiorespiratory support, pain management is crucial, as NEC is a highly painful disease.^4,5^

Since both exposure to pain and excessive analgesics (e.g., opioids) may harm preterm infants’ neurodevelopment, it is essential to provide balanced analgesic therapy to infants with NEC.^6^ However, current analgesic therapy appears not yet balanced: despite the use of paracetamol and opioids, preterm infants with NEC are unfortunately still frequently and persistently exposed to pain.^5^ This puts them at risk of the known long-term effects of neonatal pain, including impaired cognitive development, reduced growth, and behavioral problems.^7–11^ Moreover, neonatal pain may alter the development of somatosensory function (i.e., the perception of mechanosensory and noxious stimuli) and sensory processing (i.e., the integration of sensory information). For instance, procedural pain in preterm neonates has been related to lower thermal pain sensitivity and increased sensory problems (e.g., impaired auditory filtering) later in life.^12–16^ As infants with NEC not only experience daily procedural pain but also prolonged visceral pain,^17,18^ they are likely even more vulnerable to these detrimental effects.

Therefore, this study aimed to compare somatosensory function and sensory processing in preterm-born children with and without a history of NEC. Secondarily, to give a comprehensive overview, this study evaluated key self- and parent-reported outcomes, including internalizing and externalizing behavioral problems, executive functioning, chronic pain, and (health-related) quality of life.^19^ By understanding the long-term effects of NEC and associated exposure to neonatal pain and pain treatment, this study aims to enable detection of children at risk in order to provide adequate support during follow-up.

## Methods

### Study population

This cross-sectional cohort study included two groups: preterm-born children with a history of NEC (Bell’s stage II or III) and preterm-born controls without NEC, matched by sex, age, and gestational age. Children aged 6 to 15 years who had been admitted to the level III/IV neonatal intensive care unit (NICU) of the Erasmus MC – Sophia Children’s Hospital were eligible. Exclusion criteria included focal intestinal perforation without NEC (NEC diagnosis according to Bell’s diagnostic criteria^20^); insufficient understanding of the Dutch or English language to understand study information and questionnaires; severe intellectual or motor disabilities limiting the ability to participate in Quantitative Sensory Testing; and living more than a two-hour drive from the Erasmus MC – Sophia Children’s Hospital. As NEC is relatively rare, participants were included in a 1:2 ratio (NEC group vs. controls) to ensure sufficient statistical power. The institutional review board approved the study (MEC-2023-0045), and written informed consent was obtained from parents and children aged 12 years or older, with assent for children under 12. Recruitment occurred between June 2023 and October 2024. Parents of eligible participants were first informed about the study via a letter, followed by a phone call to assess their interest in participating. If they expressed interest, more information about the study was sent, and the study visit was planned. If there was no response, follow-up calls were made at two other times and a voicemail was left.

### Procedure

To make participation in the study easier, the study visit took place at the participant’s home using the Sophia research bus, a specially equipped campervan containing a clinical assessment laboratory. During the study visit, the child underwent quantitative sensory testing (QST) in the research bus and the parent completed six questionnaires. Three of these questionnaires had also been completed by children themselves in the week before the study visit.

#### Quantitative sensory testing (QST)

A shortened version of the QST protocol of the German Research Network on Neuropathic Pain was used to assess thermal detection and pain thresholds, mechanical detection threshold, and pressure pain threshold.^21,22^ All measurements were performed by a single, trained investigator (JtB).

Visual-motor reaction time for the dominant hand was assessed using open-source software, and the skin temperature of the non-dominant hand was measured with an infrared thermometer (TSR-XE200, Bintoi, China). Thermal detection and pain thresholds were measured on the thenar of the non-dominant hand using the Thermal Sensory Analyzer II (Medoc Ltd Ramat Yishai, Israel) with a 30 × 30 mm Peltier thermode, following the Method of Limits according to the protocol described by Van den Bosch et al.^23^

The mechanical detection threshold was determined using a set of 12 Von Frey filaments (Optihair, MRC Systems GmbH, Germany), applied to the dorsum of the non-dominant hand. The final threshold was the mean of five series descending and ascending stimuli.^21,22^

The pressure pain threshold was determined by applying steadily increasing pressure on the thenar of the non-dominant hand with a handheld pressure algometer (FPX50, Wagner Instruments, USA), with the mean of three series recorded as the final value.^21,22^

#### Questionnaires

Parents completed six questionnaires to evaluate their child’s sensory processing (Short Sensory Profile, SSP),^24^ behavioral problems (Child Behavior Checklist, CBCL),^25^ daily life executive function (Brief Inventory of Executive Function, BRIEF-2),^26^ chronic pain (Chronic Pain Questionnaire, CPQ),^27^ health-related quality of life (Pediatric Quality of Life Inventory, PedsQL),^28^ and subjective quality of life (Dutch-Child-AZL-TNO-Quality-of-Life, DUX25).^29^ Children aged 8 years or older were also asked to complete the CPQ, PedsQL, and DUX25 themselves via an online portal prior to the study visit. More information about these questionnaires is provided in Supplementary Table S1.

### Statistical analysis

The primary endpoints of this study were somatosensory function (mechanical/thermal detection and pain thresholds) and sensory processing (Short Sensory Profile). Secondary endpoints included internalizing (e.g., anxiety) and externalizing (e.g., aggression) behavioral problems, executive function, chronic pain, health-related quality of life, and subjective quality of life.

Preterm-born children with NEC history and controls were matched by age, sex, and gestational age using 1:4 nearest neighbor matching on propensity scores, with exact matching for sex using the R package MatchIt (Supplementary Fig. S1).^30^

Descriptive statistics were presented as mean (with standard deviation: SD), median (with inter quartile range: IQR), or number (%), as appropriate. Normality was tested using the Shapiro–Wilk test. Comparisons between the NEC group and controls were conducted using *t*-tests, Mann–Whitney U tests, chi-square tests or Fisher’s exact tests, depending on the type of data and distribution.

Multivariable linear regression was used to adjust for confounders such as age, sex, gestational age, sepsis, and infant surgery. For thermal detection and pain thresholds, truncated regression analyses were conducted to account for the baseline (32 °C), minimum (0 °C), and maximum (50 °C) temperatures. To account for potential effect modification, interaction terms between NEC and treatment type (medical vs. surgical) and NEC and sex were considered. Statistically significant interaction terms (likelihood ratio tests) were retained in the final model. Complete case analyses were performed, reporting the number of subjects per analysis.

Intraclass correlation coefficients (two-way model, absolute agreement, single measures) were used to compare child self-reported and parent-reported estimates of (health-related) quality of life.

A *p*-value of <0.05 was considered statistically significant. Given the exploratory nature of the study, no corrections for multiple testing were applied. All analyses were performed using RStudio (version 2023.12.0).

#### Sample size calculation

The required sample size was calculated for the two primary endpoints: somatosensory function (including thermal/mechanical detection and pain thresholds) and sensory processing (SSP total score). For the purpose of this calculation, heat pain threshold (HPT) was selected from the various somatosensory function outcomes as for this measure the most data were available to base the calculation on. Using two-sided *t*-tests with a significance level of 0.05, it was estimated in G*Power software that 58 children (19 NEC+, 39 NEC-) were needed to detect an 18-point difference in SSP scores, and 130 children (43 NEC+, 87 NEC-) to detect a 2 °C difference in HPT, both with 80% power.^13,16^ To reach these numbers, all eligible children with a history of NEC (*N* = 66) were invited to participate.

## Results

**Fig. 1:**
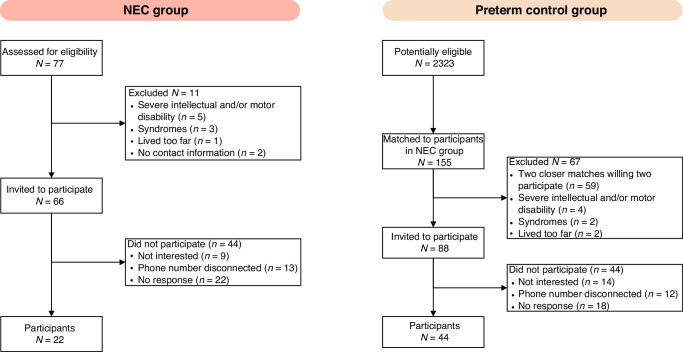
Flowchart of the inclusion of participants. Of the 66 children with a history of NEC who were invited, 22 participated. To provide two matched controls per participant in the NEC group, 88 children were invited from the control pool.

Out of 66 children with a history of NEC invited, 22 (33%) participated. To obtain 44 controls, 88 children were approached (Fig. 1). Participants’ background characteristics did not differ significantly between the two groups (Table 1). Both groups contained equal numbers of male and female participants and had a median age of 11 years. Twelve (55%) participants in the NEC group had been surgically treated. Background characteristics of participants and non-participants invited to the NEC and control groups are shown in Supplementary Table S2.

**Table 1:** Background characteristics of the participants.

	NEC (*N* = 22)	Preterm control (*N* = 44)	*P*-value
Sex: male	11 (50%)	22 (50%)	1.00
Age (years)	11 (10–13)	11 (10–14)	0.97
Gestational age (weeks)	28 (26–30)	28 (26–30)	0.96
Birth weight (grams)	1023 (874–1370)	955 (748–1218)	0.26
Birth by caesarean section	12 (55%)	21 (48%)	0.79
Apgar score 5 min after birth	8 (7–9)	8 (7–9)	0.80
Duration of NICU admission (days)	43 (20–71)	30 (11–50)	0.16
Z-score Fenton 2013 Preterm Growth Chart	0.14 (−0.46; 0.76)	−0.20 (−0.75; 0.54)	0.15
NEC stage			NA
II	15 (68%)		
III	7 (32%)		
Surgical NEC treatment	12 (55%)		NA
Other surgeries in infancy	6 (27%)	9 (20%)	0.76
Patent ductus arteriosus closure	3 (50%)	3 (33%)	
Inguinal hernia repair	4 (67%)	3 (33%)	
Other	0 (0%)	3 (33%)	
Mechanical ventilation	17 (77%)	29 (66%)	0.51
Sepsis^a^ during NICU admission	8 (36%)	11 (25%)	0.50
Received inotropics during NICU admission	8 (36%)	9 (20%)	0.27
Intraventricular hemorrhage			0.08
None	16 (73%)	41 (93%)	
Grade I	1 (5%)	1 (2%)	
Grade II	3 (14%)	1 (2%)	
Grade III	2 (9%)	1 (2%)	
Periventricular leukomalacia	2 (9%)	1 (3%)	0.26
Venous cerebral infarction	0 (0%)	0 (0%)	1.00
Retinopathy of prematurity (stage ≥ I)	12 (55%)	21 (48%)	0.79
Bronchopulmonary dysplasia (≥28 days supplemental oxygen)	14 (64%)	20 (45%)	0.20

^a^Both early and late onset sepsis, defined as positive blood culture or a negative blood culture with a CRP level > 10 mg/L within 2 days, clinical symptoms and intention to treat with antibiotics for more than five days.

Values are expressed as median (IQR) or number (%). *P*-value based on *t*-test, Mann Whitney U test, chi-square test or Fisher’s exact test, as appropriate.

### Primary endpoints

#### Somatosensory function

**Fig. 2:**
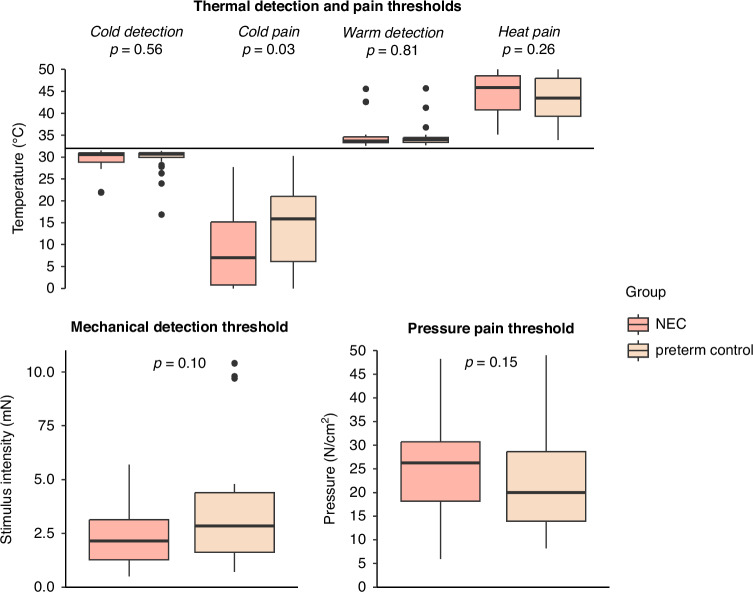
Quantitative sensory testing results of the NEC group and preterm control group. Mann–Whitney U tests revealed no significant differences between the NEC group and preterm control group, except for a significantly lower cold pain threshold in the NEC group.

All 66 participants completed QST. Cold pain threshold was at a significantly lower temperature in the NEC group (indicating lower pain sensitivity), as identified by univariable (*p* = 0.03) and multivariable analyses (*β* = −11 °C, 95% CI [−21; −1.0], *p* = 0.03). All other thresholds did not differ significantly between the two groups (Fig. 2). During cold pain threshold assessment, 41% of the NEC group and 23% of the control group reached the minimum temperature of 0 °C at least once. During heat pain threshold assessment, 32% of both groups reached the maximum temperature of 50 °C.

In the overall group, male sex and older age were associated with a lower cold pain threshold and a higher heat pain threshold (Table 2). Children surgically treated for NEC had on average lower cold pain thresholds (6.2 vs. 13 °C) and higher heat pain thresholds (46 vs. 44 °C) than those conservatively treated for NEC, but these differences were not statistically significant.

**Table 2:** Results of the multivariable linear regression analyses: quantitative sensory testing.

	CDT		WDT		CPT		HPT		MDT		PPT	
	*β (95% CI)*	*P-value*	*β (95% CI)*	*P-value*	*β (95% CI)*	*P-value*	*β (95% CI)*	*P-value*	*β (95% CI)*	*P-value*	*β (95% CI)*	*P-value*
Intercept	22		45		58		21		432		13	
NEC	−0.05 (−1.7; 0.80)	0.47	0.33 (−0.89; 1.5)	0.59	−11 (−21; −1.0)	**0.03**	1.3 (−0.96; 3.64)	0.25	−15 (−73; 43)	0.61	2.5 (−2.8; 7.8)	0.35
Age	0.19 (−0.07; 0.45)	0.15	−0.28 (−0.53; −0.04)	**0.03**	−2.0 (−3.9; −0.13)	**0.04**	0.91 (0.45; 1.4)	**<0.001**	−3.6 (−16; 8.4)	0.55	1.1 (−0.02; 2.2)	0.05
Male sex	0.48 (−0.78; 1.7)	0.45	−0.43 (−1.6; 0.77)	0.49	−11 (−20; −1.9)	**0.02**	2.7 (0.52; 4.9)	**0.01**	52 (−5.5; 110)	0.08	4.9 (−0.39; 10)	0.07
Gestational age	0.18 (−0.11; 0.47)	0.22	−0.24 (−0.51; 0.04)	0.09	−0.67 (−2.5; 1.2)	0.49	0.38 (−0.13; 0.88)	0.16	−13 (−27; −0.11)	**0.05**	−0.19 (−1.4; 1.0)	0.75
Sepsis	−0.28 (−1.7; 1.2)	0.70	0.33 (−1.0; 1.7)	0.63	−1.7 (−11; 7.8)	0.72	−0.24 (−2.7; 2.2)	0.85	0.47 (−66; 67)	0.99	1.0 (−5.0; 7.0)	0.74
Infant surgery (other than NEC)	0.44 (−1.0; 1.9)	0.60	−0.72 (−2.1; 0.70)	0.32	−2.8 (−13; 7.6)	0.59	−0.09 (−2.6; 2.4)	0.94	−6.6 (−75; 62)	0.85	−1.2 (−7.4; 5.0)	0.70

*CDT* cold detection threshold (°C), *WDT* warm detection threshold (°C), *CPT* cold pain threshold (°C), *HPT* heat pain threshold (°C), *MDT* mechanical detection threshold (mN), *PPT* pressure pain threshold (N/cm^2^).

Significant *P*-values are indicated in bold.

Reaction times and skin temperature did not differ significantly between the NEC group and the control group (*p* = 0.34 and *p* = 0.82, respectively), and were therefore not taken into account in further analyses.

#### Sensory processing

**Fig. 3:**
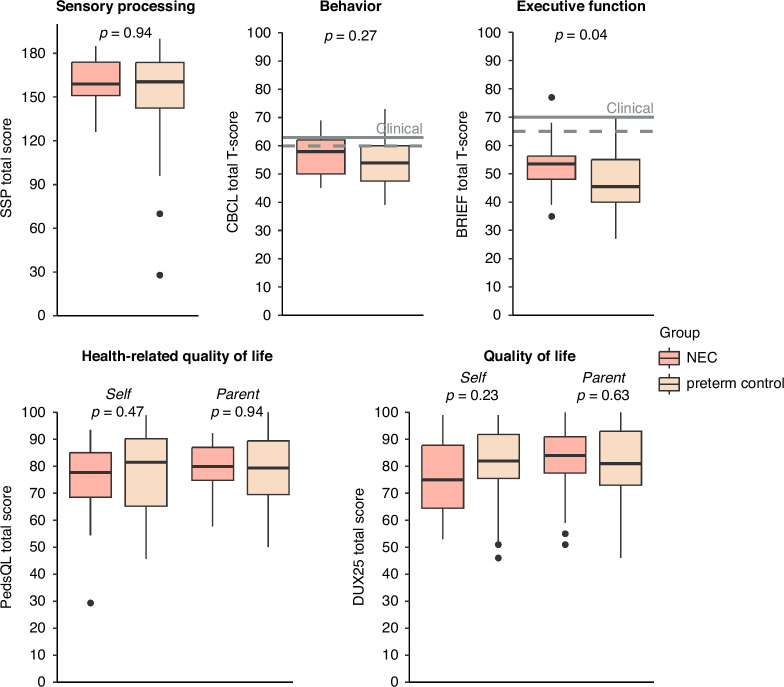
Main questionnaire results of the NEC group and preterm control group. T-tests and Mann–Whitney U tests identified no significant differences between the two groups, except for a significantly higher BRIEF2 score in the NEC group.

Parents of 21 (95%) children in the NEC group and 42 (95%) in the preterm control group completed the SSP questionnaire. The SSP total score did not differ significantly between the two groups (Fig. 3), nor was it associated with any of the other participant characteristics (Table 3). Eleven (52%) children in the NEC group and 21 (50%) in the preterm control group scored in the atypical range (−2 SD from reference values) for at least one of the sensory domains. In both groups, sensory processing problems were particularly common in the tactile sensitivity and auditory filtering domains (Supplementary Fig. S2).

**Table 3:** Results of the multivariable linear regression analyses: questionnaires.

	SSP		CBCL		BRIEF		PedsQL^a^		DUX25^a^	
	*β (95% CI)*	*P-value*	*β (95% CI)*	*P-value*	*β (95% CI)*	*P-value*	*β (95% CI)*	*P-value*	*β (95% CI)*	*P-value*
Intercept	197		32		28		47		154	
NEC	8.0 (−8.1; 24)	0.32	2.5 (−2.1; 7.1)	0.28	6.1 (−0.25; 12)	0.06	−3.6 (−12; 4.5)	0.38	−3.1 (−10; 4.0)	0.39
Age	2.5 (−0.82; 5.8)	0.14	−0.15 (−1.1; 0.81)	0.75	−0.42 (−1.8; 0.92)	0.53	0.60 (−1.1; 2.3)	0.49	−1.7 (−3.2; −0.16)	**0.03**
Male sex	−4.2 (−20; 11)	0.59	−1.9 (−6.4; 2.3)	0.40	−3.3 (−9.7; 3.0)	0.30	2.0 (−6.1; 10)	0.62	0.61 (−6.6; 7.8)	0.87
Gestational age	−2.6 (−6.3; 1.0)	0.15	0.91 (−0.15; 2.0)	0.09	0.98 (−0.50; 2.5)	0.20	0.24 (−1.6; 2.1)	0.80	−2.1 (−3.8; −0.41)	**0.02**
Sepsis	6.5 (−12; 24)	0.47	−2.2 (−7.4; 3.0)	0.40	−4.4 (−11; 2.6)	0.21	5.1 (−3.9; 14)	0.26	9.0 (0.95; 17)	**0.03**
Infant surgery (other than NEC)	5.3 (−14; 25)	0.58	−0.63 (−6.1; 4.9)	0.82	−0.14 (−7.6; 7.4)	0.97	−4.1 (−13; 5.1)	0.38	−5.5 (−14; 3.1)	0.20

^a^If available based on self-report, otherwise based on parent-report.

*SSP* short sensory profile, *CBCL* child behavior checklist, *BRIEF* behavior rating inventory of executive function; *PedsQL* pediatric quality of life inventory, *DUX25* Dutch-Child-AZL-TNO-Quality-of-Life.

Significant *P*-values are indicated in bold.

### Secondary endpoints

#### Behavior

The CBCL questionnaire was completed for 21 (95%) and 40 (91%) participants in the NEC and control group, respectively. No significant differences were found in T-score for total problems (*p* = 0.27), internalizing problems (*p* = 0.82), and externalizing problems (*p* = 0.20). Multivariable regression analysis found no significant associations with total T-score either (Table 3). On the total problems scale, four (19%) children in the NEC group and six (15%) in the preterm control group had behavioral problems in the clinical range (+2 SD from mean) (Supplementary Fig. S3). On the internalizing problems scale, seven (33%) children in the NEC group and 15 (38%) in the control group had problems in the clinical range. On the externalizing problems scale, two (10%) children in the NEC group and two (5%) in the control group had problems in the clinical range.

#### Executive function

The BRIEF2 questionnaire was completed for 20 (91%) and 34 (77%) participants in the NEC group and control group, respectively. Univariable analysis showed a significantly higher total T-score in the NEC group (*p* = 0.03), indicating poorer executive functioning (Fig. 3). The NEC group had a higher behavior regulation index score (*p* = 0.02), while the emotion regulation (*p* = 0.07) and cognitive regulation (*p* = 0.18) indices did not differ significantly between the two groups. Overall, executive function was classified as clinical in two (10%) children in the NEC group and one (3%) in the preterm control group (Supplementary Fig. S4). In multivariable analysis, the association between NEC and total T-score was no longer significant (*β* = 6.1, 95% CI [−0.25; 12], *p* = 0.06) (Table 3).

#### Chronic pain

Of the children aged 8 years or older, 20 in the NEC group and 39 in the preterm control group completed the CPQ. Since the parent-reported results (20 in NEC group, 34 in preterm control group) were similar to the self-reported results, only the self-reported results are presented here.

Seven (35%) children in the NEC group and 17 (44%) in the preterm control group reported pain in the past three months, of whom two (10%) children in the NEC group and six (15%) in the control group reported chronic pain (i.e., longer than three months). The prevalence of acute pain and chronic pain did not differ significantly between the two groups (*p* = 0.72 and p = 0.74, respectively), nor did the reported pain intensity, with a mean score of 39 (SD 14) out of 100 in the NEC group and 42 (SD 20) in the preterm group (*p* = 0.60). In both groups, the most reported pain locations were the stomach, head, arms and legs, and ear. When in pain, most children continue with their activities at a slower pace (Supplementary Fig. S5).

#### Health-related quality of life

The PedsQL questionnaire was completed by 20 children and 20 parents in the NEC group and 39 children and 32 parents in the control group. There were no significant differences in self- or parent-reported total T-scores between the two groups (*p* = 0.47 and *p* = 0.94, respectively). Multivariable linear regression analysis found no significant associations either (Table 3).

Agreement between self- and parent-reported scores was moderate for total score (ICC 0.61), physical functioning (ICC 0.60), emotional functioning (ICC 0.57), and school functioning (ICC 0.59), but poor for social functioning (ICC 0.29). Supplementary Figs. S6 and S7 show total and subscale scores in the two groups and the correlations between self- and parent-reported scores.

#### Quality of life

DUX25 questionnaires were completed by 20 children and 19 parents in the NEC group and 37 children and 33 parents in the control group. There were no significant differences between participants whose parents completed all questionnaires and those with missing questionnaires, except for a lower gestational age observed in the three children within the NEC group whose parents did not complete all questionnaires (Supplementary Table S3). Self- and parent-reported DUX25 total T-scores did not differ significantly between the two groups (*p* = 0.23 and *p* = 0.63, respectively). Multivariable analysis found no significant association between DUX25 total T-score and NEC either, but did find that older age (*β* = −1.7, 95% CI [−3.2; −0.16], *p* = 0.03) and a higher gestational age (*β* = −2.1, 95% CI [−3.8; −0.41], *p* = 0.02) were associated with worse, and neonatal sepsis (*β* = 9.0, 95% CI 0.95; 17], *p* = 0.03) with better quality of life (Table 3).

Agreement between self- and parent-reported DUX25 scores was good for total score (ICC 0.82) and moderate for home functioning (ICC 0.61), emotional functioning (ICC 0.74), and social functioning (ICC 0.71) scores. Supplementary Figs. S8 and S9 show total and subscale scores in the two groups and the correlations between self- and parent-reported scores.

## Discussion

This cross-sectional cohort study shows that compared to other preterm-born children, children with a history of the painful intestinal disease NEC have lower sensitivity for cold pain, but apart from that similar somatosensory function (detection and pain thresholds) and sensory processing – our primary endpoints. Furthermore, daily life executive functioning was poorer in those with NEC history, although this difference was no longer significant after adjustment for clinical factors. Other self- and parent-reported outcomes, including internalizing and externalizing behavioral problems, chronic pain, and (health-related) quality of life did not differ significantly between those with and without NEC history. These findings highlight that, while NEC has lasting impacts in specific areas, many outcomes for children with NEC history resemble those of other preterm-born children.

The lower cold pain threshold in the NEC group aligns with previous studies using the TSA, which found reduced thermal pain sensitivity in preterm children with NICU experience, particularly those who had neonatal surgery such as patent ductus arteriosus closure or inguinal hernia repair.^13–15,31^ Contrarily, studies using the Cold Pressor Task have found increased pain sensitivity in children with NICU experience, including those with NEC.^14,32^ This discrepancy may be due to more prolonged cold stimulation during the Cold Pressor Task. Supporting this, studies found that although children with NICU experience had higher heat pain thresholds, they exhibited less habituation (i.e., greater perceptual sensitization) to tonic heat.^14,15,31^ Regarding thermal detection, mechanical detection, and pressure pain thresholds, prior studies reported mixed results. Some indicate decreased sensitivity in those with (greater) neonatal pain, while others, including our study, found no significant differences.^13–15,33,34^ However, the results of previous studies are not directly comparable to ours, as most studies included the general NICU population, similar to our control group, whereas infants with NEC represent a unique population of generally very preterm infants exposed to both procedural and prolonged visceral pain. Compared to reference values for healthy Dutch children, our study found similar detection and heat pain thresholds, but cold pain thresholds appear higher in the preterm control group (i.e., more sensitive) and lower in the NEC group (i.e., less sensitive).^23^

Modality-specific changes in somatosensory function may be explained by the involvement of different nerve fibers: Aβ fibers for mechanical detection, Aδ fibers for cold detection, C fibers for warm detection and heat pain, and both C and Aδ fibers for cold and pressure pain.^22^ Alternatively, the sample size may have been insufficient to identify changes in heat and pressure pain thresholds (type 2 error) or the altered cold pain threshold could be due to type 1 error. Reduced cold pain sensitivity may result from increased inhibitory output from the descending pain modulatory system, as a compensatory response to neonatal pain.^35–37^ Enhanced perceptual sensitization during prolonged stimulation,^14,15,31^ on the other hand, suggests central sensitization of nociceptive pathways that may no longer be compensated by increased descending inhibition.^35,37^ These long-term effects may depend on the balance between pain and analgesic treatment during infancy, as sufficient pain management may mitigate such changes.^6^ Previous studies have shown that pre-emptive analgesic therapy attenuates long-term changes in pain responsivity after infant surgery.^38,39^ Preclinical research found that the ability to prevent long-term changes in somatosensory function after neonatal pain varied between analgesia techniques, suggesting optimal analgesia is key to prevent long-term changes.^40^

Besides central sensitization, hypersensitivity may arise from peripheral sensitization at sites of prior neonatal injury.^6,37,41^ However, in this study, there were fortunately no signs that those who had NEC were more sensitive to abdominal pain, though this is based on reported stomachache rather than objective visceral pain sensitivity measurements. Beyond biological factors, environmental and psychosocial influences, such as maternal presence or maladaptive coping strategies, can influence pain responses.^14,31,32^ For this reason, parents were allowed to be present during the study visit but remained out of the child’s sight to avoid influencing pain thresholds.

Atypical sensory processing was common among our study participants, as previously reported in preterm children,^42–44^ but sensory processing did not differ between those with and without NEC history. Although sensory processing has not been studied before in children with a history of NEC, altered sensory processing may be expected in these children based on previous studies linking neonatal pain exposure with atypical sensory processing.^12,16^ However, preterm neonates admitted to the NICU are exposed to various developmentally unexpected sensory stimuli (e.g., sounds, lights) that may disturb the development of sensory processing,^45^ of which pain is just one, and they are all exposed to painful procedures.

Our finding of poorer parent-reported daily life executive function in the NEC group is consistent with two previous studies,^46,47^ while two other studies found no significant differences between preterm-born children with and without NEC history.^48,49^ These mixed results in previous studies are dependent on measurement materials (parent report vs. task performance) and domain of executive functioning measured (attention vs. memory vs. mental flexibility vs. planning). Impaired executive function in terms of behavioral regulation in children with a history of NEC, as found in the current study, may be attributed to their increased risk of developing white matter abnormalities,^50^ which in turn have been associated with impaired executive function later in life.^51^ However, clinical problems in executive function were rare in both groups. By contrast, internalizing problems were prevalent, with approximately a third exhibiting clinical problems, though the occurrence of behavioral problems did not differ significantly between the two groups, consistent with previous studies.^48,52,53^ These findings suggest that monitoring of behavioral (regulation) problems in children with a history of NEC is of importance, with a specific focus on internalizing and externalizing behavioral problems in all preterm-born children, independent of NEC history. Monitoring from early life will make it possible to determine early markers of ‘at risk’ developmental profiles, creating opportunities for early intervention.

Acute and chronic pain prevalences were similar in the two groups, and slightly lower than those reported in healthy Dutch children.^27^ Contrarily to some studies reporting impaired quality of life in (surgical) NEC survivors,^54,55^ our study found no significant differences in (health-related) quality of life between the two groups, with PedsQL scores in the same range as those of healthy Dutch children.^28^ However, scores varied considerably, especially in emotional functioning. Agreement between self- and parent-reported (health-related) quality of life was generally moderate but poorer for social functioning, consistent with previous findings.^56^

The finding that children with a history of NEC show changes in cold pain thresholds even years later may highlight the importance of effective analgesic therapy for these infants, as the pain management they received may have been insufficient to prevent these changes. However, the underlying mechanisms remain speculative, and other pain thresholds were not significantly altered. It is reassuring that chronic pain prevalence did not differ between preterm-born children with and without NEC history. Likewise, the lack of significant differences in sensory processing, behavior, and (health-related) quality of life between the two groups is reassuring, although in both groups atypical sensory processing and internalizing problems were common. The finding of poorer executive function in the NEC group warrants caution, though fortunately most children in both groups had normal executive function. Nonetheless, thorough follow-up of preterm-born infants is necessary to identify any issues and provide timely support.

A major strength of this study is its focus on a previously unexplored topic – somatosensory function and sensory processing in children who had NEC – alongside key outcomes such as quality of life. This has resulted in a comprehensive overview of the outcomes of preterm-born children with and without NEC history, in contrast with previous studies which typically focused on a specific domain. Moreover, also asking children themselves to complete the questionnaires on chronic pain and (health-related) quality of life enabled us to capture their unique perspectives. A limitation is that only a subset of eligible participants could be reached, potentially limiting the power and generalizability of our findings. Moreover, the most severely affected children – those unable to participate due to cognitive and/or motor impairments – were not included. Additionally, our sample size, though comparable to previous QST studies in preterm-born children, was likely too small to examine interactions between NEC history and factors like sex and type of NEC treatment (surgical vs. conservative), and no adjustments were made for multiple testing. Based on our sample size calculation, especially the analyses of thermal pain thresholds may have been underpowered. Moreover, it was not possible to blind the investigator to participants’ group status (NEC vs. preterm control), as the investigator performed a scar inspection in those surgically treated for NEC (data presented in a separate manuscript). However, standardized instructions during QST ensured that the results were not influenced, and the questionnaires were completed online independently of the investigator. Another limitation is that no individual data on participants’ exposure to pain and opioids during NICU admission were available, limiting our ability to draw conclusions about the effects of pain and opioid exposure on our endpoints. However, exposure to pain and opioids during NEC have already been described in a previous retrospective study at our NICU, which showed that NEC leads to persistent and prolonged periods of pain and requires systemic opioids for considerable amounts of time.^5^ Besides pain and pain treatment, a variety of other factors may influence long-term outcomes after NEC, such as the excessive inflammation, altered feeding, and critical illness in infants with NEC, making it difficult to disentangle causal effects of different factors. This applies especially to complex endpoints such as executive function, whereas changes in pain thresholds are likely more directly related to pain exposure during NICU admission.

## Conclusions

Preterm-born children with a history of NEC exhibit reduced cold pain sensitivity but otherwise similar somatosensory function and sensory processing compared to other preterm-born children. They may experience poorer executive function, while behavior, chronic pain, and (health-related) quality of life outcomes are comparable to those of other preterm-born children. Although these results are generally reassuring, the altered cold pain sensitivity after such a long period of time underlines the critical importance of adequate pain management during NEC. Additionally, the high prevalence of sensory processing problems and behavioral problems in both groups emphasizes the need for thorough follow-up after NICU discharge for all very preterm-born infants.

## Supplementary information

Supplementary material

## Data Availability

The datasets generated during and/or analyzed during the current study are available from the corresponding author on reasonable request.
