# Supplementary material for: Long-term impact of necrotizing enterocolitis on somatosensory function in preterm born children
Source: Pediatr Res. 2025 Aug 26;99(3):1113–21. doi: 10.1038/s41390-025-04348-8 (PMC13021523; doi:10.1038/s41390-025-04348-8)

Contents

Supplementary table S1. Information about the used questionnaires

Supplementary figure S1. Covariate balance before and after matching

Supplementary table S2. Characteristics of those included and those invited but not included

Supplementary table S3. Characteristics of participants whose parents completed all questionnaires and those with missing questionnaires

Supplementary figure S2. Classification of sensory processing

Supplementary figure S3. Classification of behavior

Supplementary figure S4. Classification of executive function

Supplementary figure S5. Self-reported behavioral responses to pain

Supplementary figure S6. PedsQL total score and subscale scores

Supplementary figure S7. Correlation between self- and parent-reported PedsQL scores

Supplementary figure S8. DUX25 total score and subscale scores

Supplementary figure S9. Correlation between self- and parent-reported DUX25 scores

Supplementary table S1. Information about the used questionnaires

Questionnaire	Domains	Score range and interpretation	Development and validation
Short Sensory Profile (SSP)	Tactile sensitivity; Taste/smell sensitivity; Movement sensitivity; Underresponsive/ seeks sensation; Auditory filtering; Low energy/weak; Visual/auditory sensitivity	Sum scores for each section are compared with normative data to classify sensory processing as typical, possibly atypical (-1 SD) or definitely atypical (-2 SD). Lower scores indicate greater sensory processing difficulties. Total scores range from 38 to 190.	McIntosh et al [1]<br><br>Dutch reference values available.
Child Behavior Checklist (CBCL)	Problem scales:<br>Internalizing problems; Externalizing problems; Total problems<br><br>DSM-V scales:<br>Affective problems; Anxiety problems; Attention deficit/ hyperactivity problems; Oppositional/defiant; Behavioral problems<br><br>Syndrome scales:<br>Anxious/depressed; Withdrawn/depressed; Somatic complains; Social problems; Thought problems; Attention problems; Rule-breaking behavior; Aggressive behavior	Raw scores are converted into T-scores (0-100), with higher scores indicating greater problems. T-scores ≥ 60 are classified as borderline clinical and T-scores ≥ 63 as clinical.	Achenbach & Edelbrock [2]<br><br>Dutch reference values available.
Brief Inventory of Executive Function (BRIEF-2)	Scales:<br>Inhibit; Self-monitor; Shift; Emotional control; Initiate; Working memory; Plan/organize; Task-monitor<br><br>Indexes:<br>Behavioral regulation index; Emotional regulation index; Cognitive regulation index	Raw scores are converted into T-scores (0-100), with higher scores indicating greater problems. T-scores ≥ 65 are classified as potentially clinically elevated and T-scores ≥ 70 as clinically elevated.	Gioia et al [3]<br><br>Psychometric properties[4]<br><br>Dutch reference values available.
Chronic Pain Questionnaire (CPQ)	Pain intensity; Pain duration; Pain frequency; Pain interference with daily activities	The questionnaire provides estimates of pain occurrence and burden in the past three months.	Perquin et al [5]
Pediatric Quality of Life Inventory (PedsQL)	Physical functioning; Emotional functioning; Social functioning; School functioning; Psychosocial health summary; Physical health summary; Total score	Raw scores are converted into T-scores (0-100), with higher scores indicating better health-related quality of life.	Varni et al [6]<br><br>Psychometric properties Dutch version[7]<br><br>Dutch reference values available.[8]
Dutch-Child-AZL-TNO-Quality-of-Life (DUX25)	Physical functioning; Emotional functioning; Social functioning; Home functioning; Total functioning	Raw scores are converted into T-scores (0-100), with higher scores indicating better quality of life.	Vogels et al [9]<br><br>Dutch reference values available.

[1] McIntosh DN, Miller LJ, Shyu V, Dunn W. Development and validation of the short sensory profile. Sensory profile manual. 1999;61:59-73.
[2] Achenbach TM, Edelbrock C. Child behavior checklist. Burlington (Vt). 1991;7:371-92.
[3] Gioia GA, Isquith PK, Guy SC, Kenworthy L. BRIEF-2: Behavior rating inventory of executive function: Psychological Assessment Resources Lutz, FL; 2015.

[4] Hendrickson NK, McCrimmon AW. Test Review: Behavior Rating Inventory of Executive Function®, (BRIEF® 2) by Gioia, GA, Isquith, PK, Guy, SC, & Kenworthy, L. SAGE Publications Sage CA: Los Angeles, CA; 2019.
[5] Perquin CW, Hazebroek-Kampschreur A, Hunfeld JAM, Bohnen AM, van Suijlekom-Smit LWA, Passchier J, van der Wouden JC. Pain in children and adolescents: a common experience. Pain. 2000;87:51-8.
[6] Varni JW, Seid M, Kurtin PS. PedsQL 4.0: reliability and validity of the Pediatric Quality of Life Inventory version 4.0 generic core scales in healthy and patient populations. Med Care. 2001;39:800-12.
[7] Engelen V, Haentjens MM, Detmar SB, Koopman HM, Grootenhuis MA. Health related quality of life of Dutch children: psychometric properties of the PedsQL in the Netherlands. BMC Pediatr. 2009;9:68.
[8] van Muilekom MM, Luijten MAJ, van Oers HA, Conijn T, Maurice-Stam H, van Goudoever JB, et al. Paediatric patients report lower health-related quality of life in daily clinical practice compared to new normative PedsQL(TM) data. Acta Paediatr. 2021;110:2267-79.
[9] Vogels T, Verrips GH, Verloove-Vanhorick SP, Fekkes M, Kamphuis RP, Koopman HM, et al. Measuring health-related quality of life in children: the development of the TACQOL parent form. Qual Life Res. 1998;7:457-65.

Supplementary figure S1. Covariate balance before and after matching

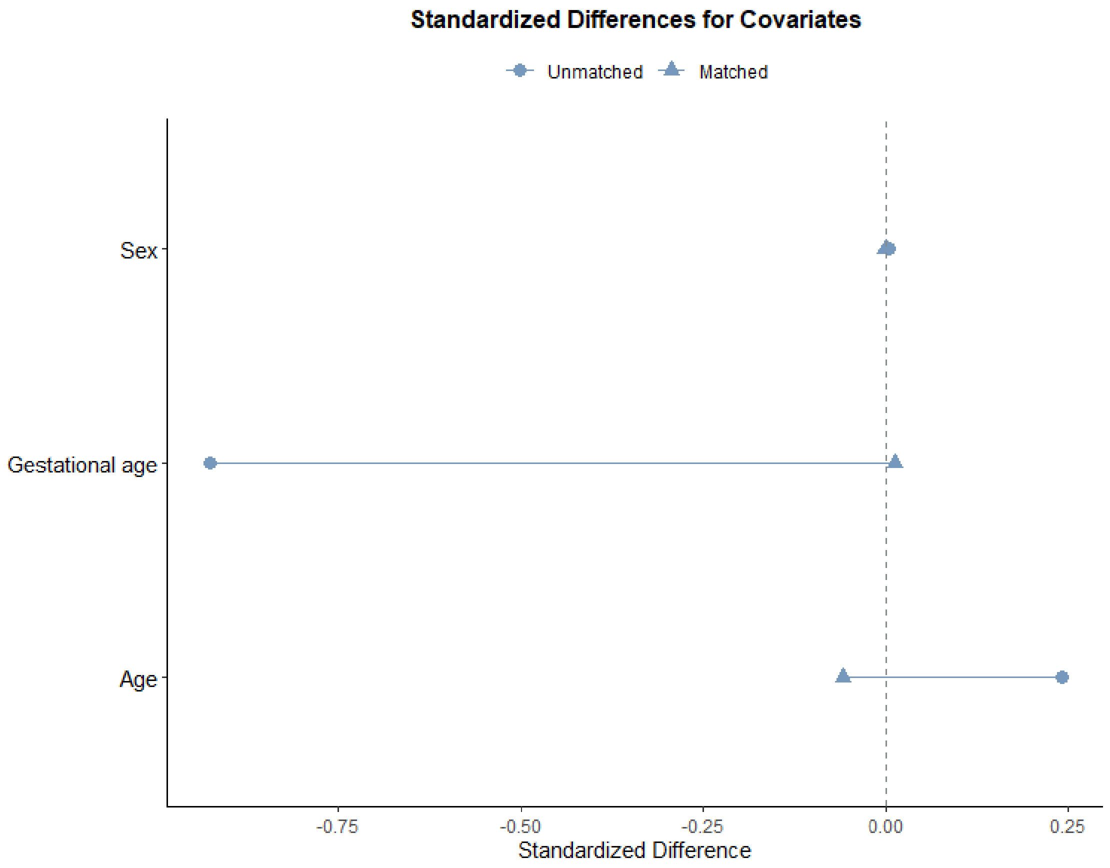

Supplementary table S2. Characteristics of those included and those invited but not included

	**NEC**			**Preterm control**		
	Included (N=22)	*Not included (N=44)*	*P-value*	*Included (N=44)*	*Not included (N=44)*	*P-value*
Sex: male	11 (50%)	24 (55%)	0.93	22 (50%)	33 (75%)	0.03
Age (years)	11 (10-13)	11 (9-14)	1.00	11 (10-14)	12 (10-15)	0.29
Gestational age (weeks)	28 (26-30)	27 (26-28)	0.10	28 (26-30)	28 (26-29)	0.73
Birth weight (grams)	1023 (874-1370)	885 (695-1065)	0.03	955 (748-1218)	1023 (821-1218)	0.41
Birth by caesarean section	12 (55%)	26 (59%)	1.00	21 (48%)	27 (61%)	0.28
Apgar score 5 minutes after birth	8 (7-9)	8 (7-9)	0.73	8 (7-9)	8 (7-9)	0.76
Duration of NICU admission (days)	43 (20-71)	60 (35-91)	0.16	30 (11-50)	26 (16-52)	0.91
Surgical NEC treatment	12 (55%)	25 (57%)	0.93			

Values are expressed as median (IQR) or number (%). P-value based on t-test, Mann Whitney U test, chi-square test or Fisher's exact test, as appropriate.

Supplementary table S3. Characteristics of participants whose parents completed all questionnaires and those with missing questionnaires

	**NEC**			**Preterm control**		
	Complete (N=19)	*Incomplete (N=3)*	*P-value*	*Complete (N=33)*	*Incomplete (N=11)*	*P-value*
Sex: male	9 (47%)	2 (66%)	1.00	17 (55%)	5 (45%)	1.00
Age (years)	11 (10-12)	14 (13-15)	0.12	11 (10-14)	12 (10-14)	0.75
Gestational age (weeks)	28 (27-30)	25 (25-26)	0.02	28 (26-30)	28 (27-31)	0.44
Birth weight (grams)	1140 (908-1395)	870 (805-878)	0.08	980 (825-1190)	875 (683-1350)	0.59
Birth by caesarean section	11 (58%)	1 (33%)	0.87	15 (45%)	6 (55%)	0.86
Apgar score 5 minutes after birth	8 (7-9)	7 (7-8)	0.35	8 (6-9)	8 (7-9)	0.58
Duration of NICU admission (days)	36 (18-65)	68 (67-103)	0.11	31 (12-58)	28 (5-41)	0.27
Surgical NEC treatment	11 (58%)	1 (33%)	0.87			

Values are expressed as median (IQR) or number (%). P-value based on t-test, Mann Whitney U test, chi-square test or Fisher's exact test, as appropriate.

Supplementary figure S2. Classification of sensory processing

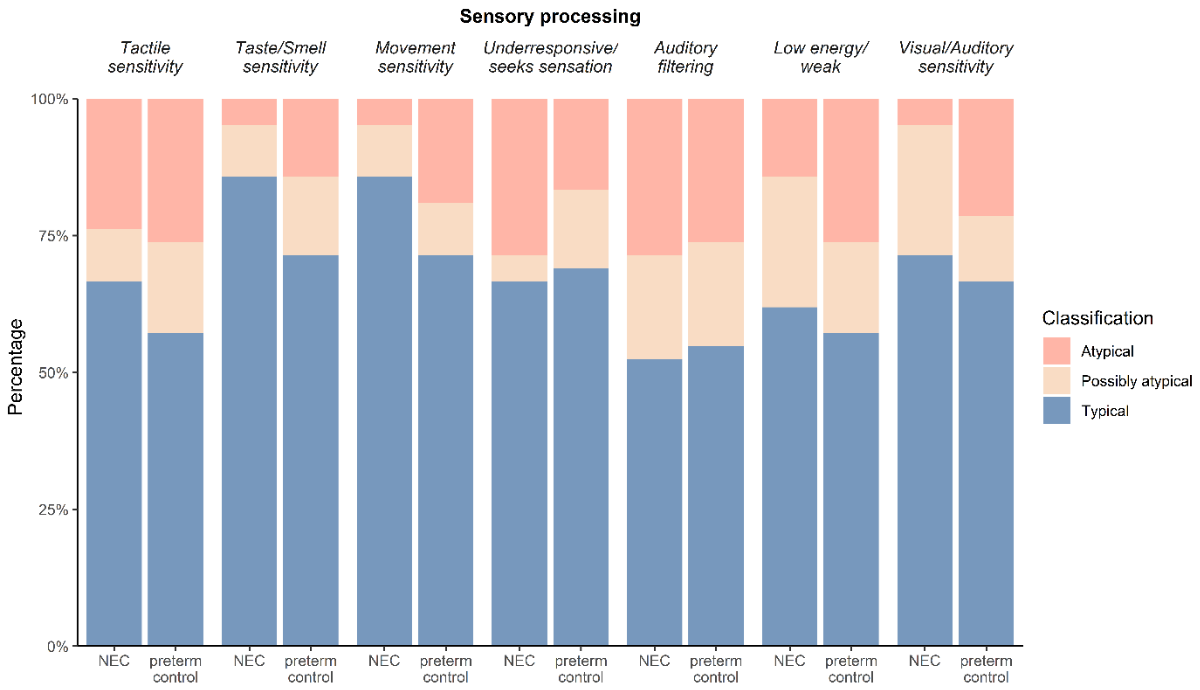

Supplementary figure S3. Classification of behavior

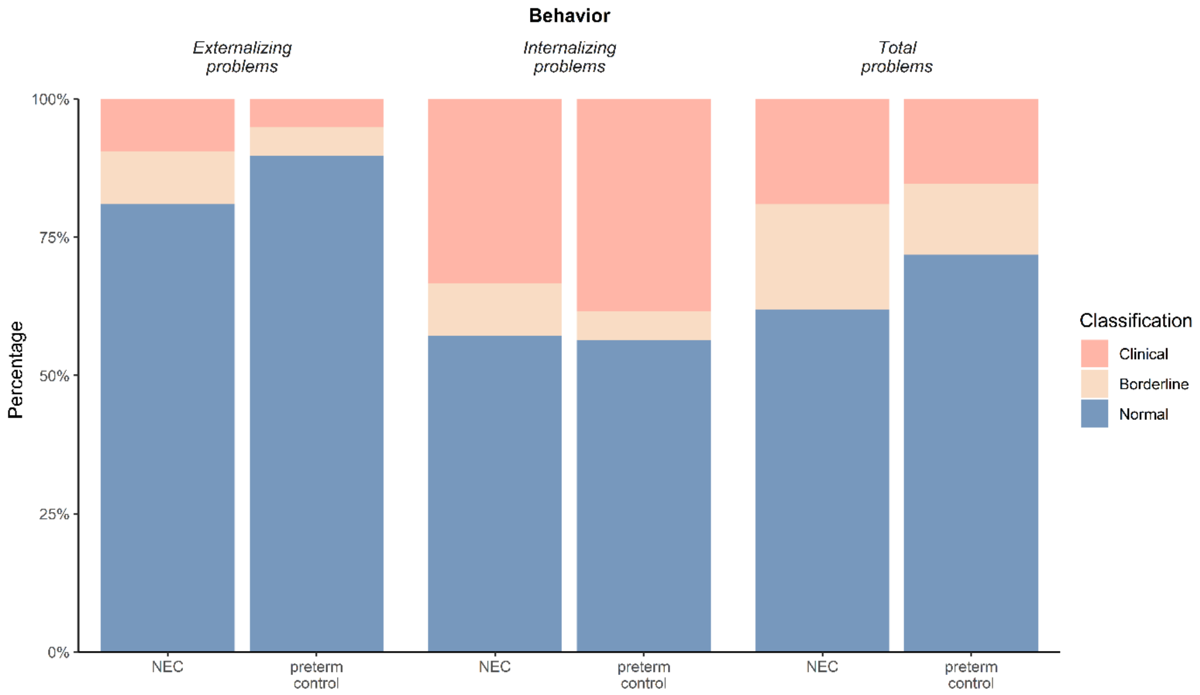

Supplementary figure S4. Classification of executive function

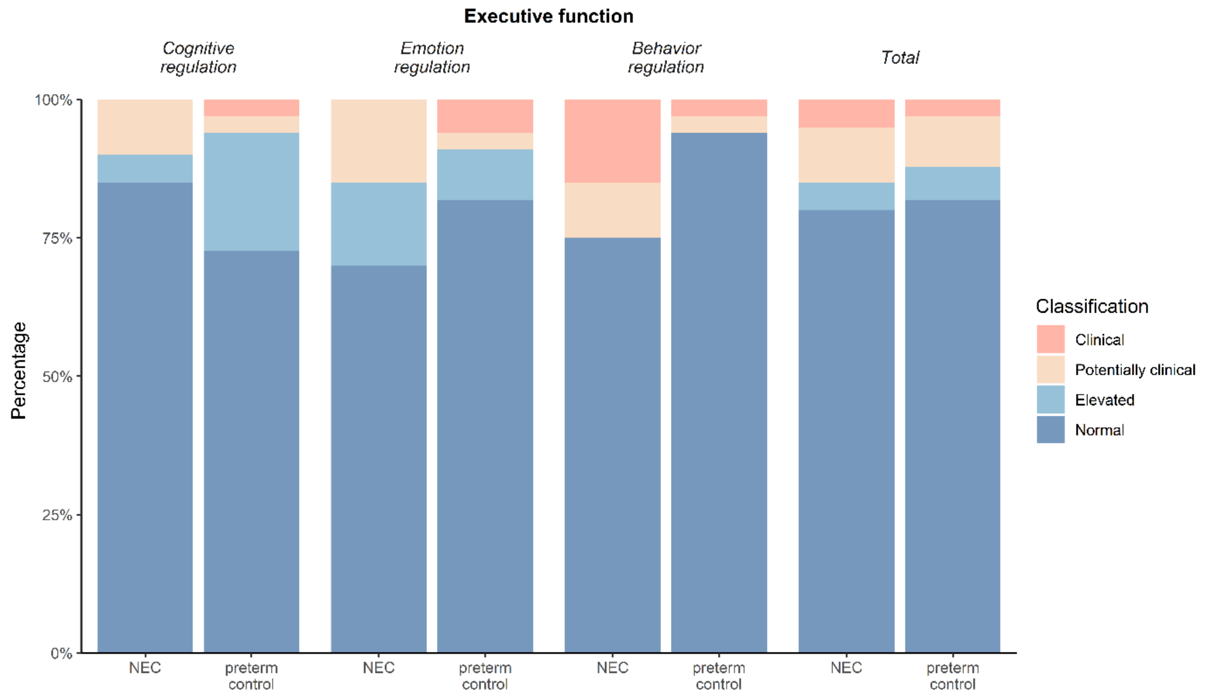

Supplementary figure S5. Self-reported behavioral responses to pain

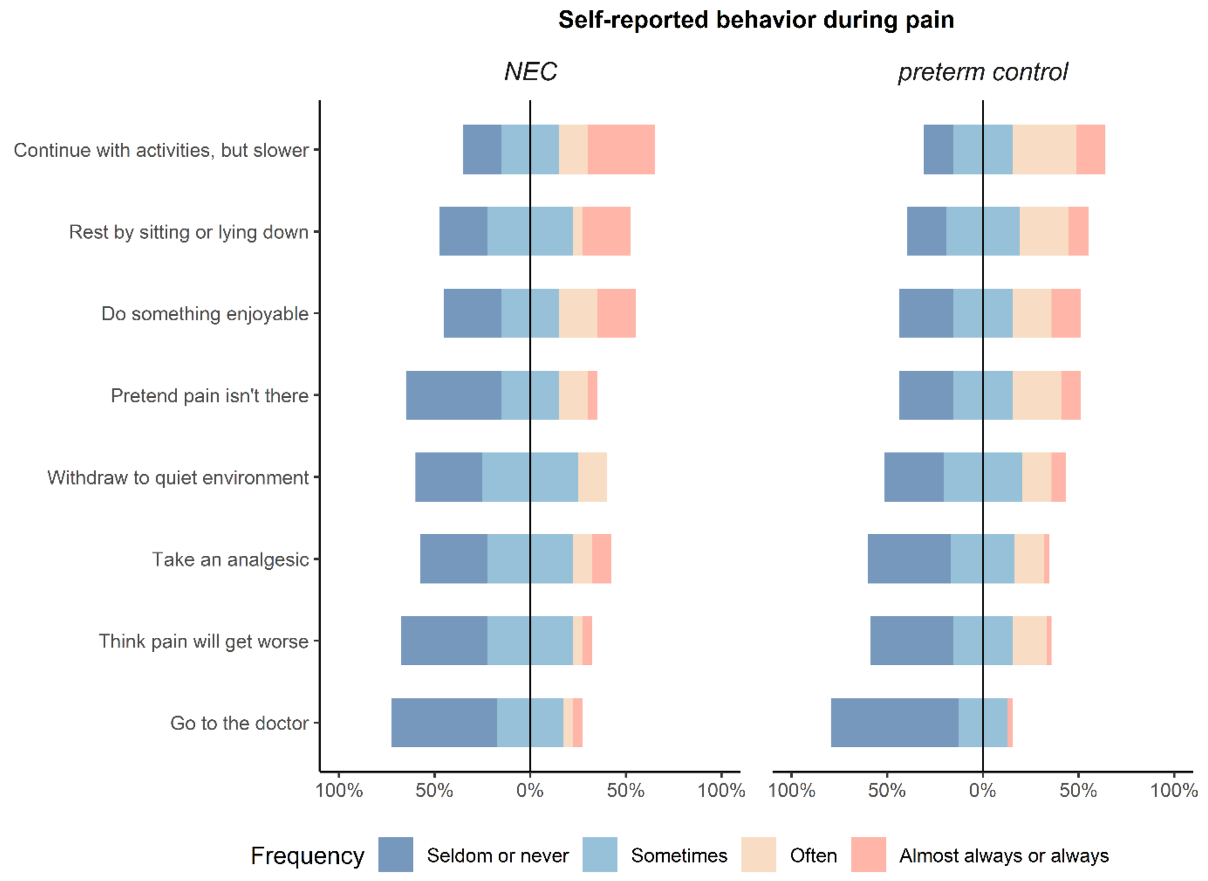

Supplementary figure S6. PedsQL total score and subscale scores

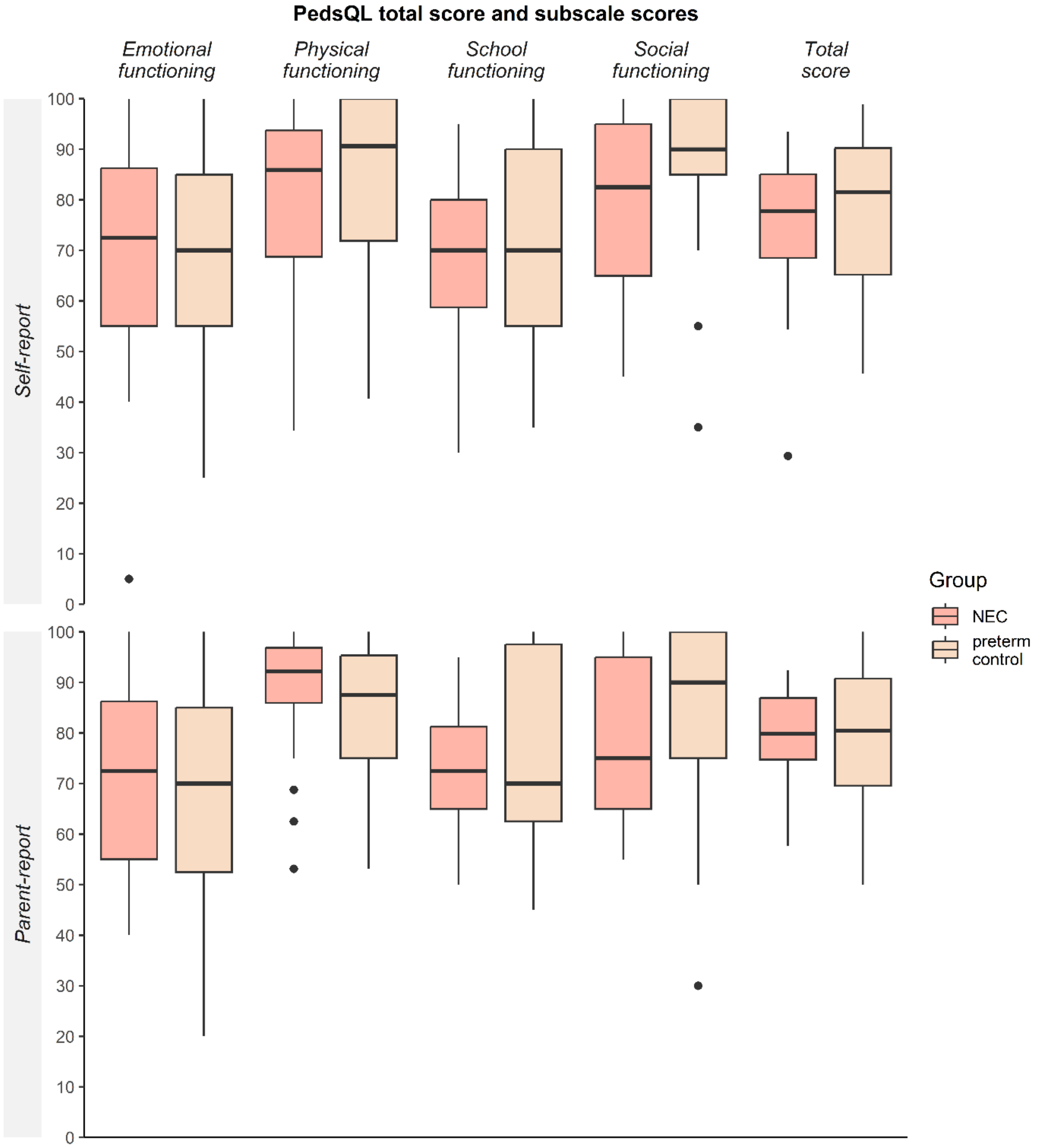

Supplementary figure S7. Correlation between self- and parent-reported PedsQL scores

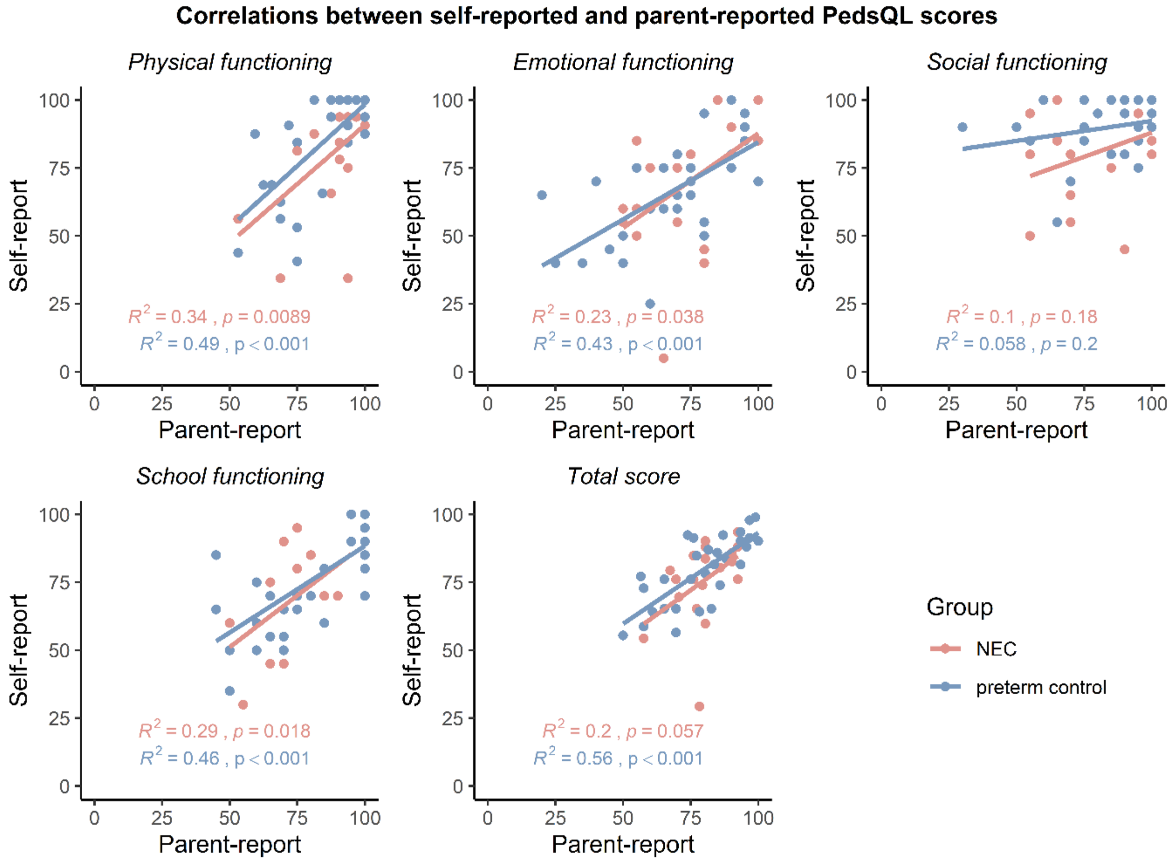

Supplementary figure S8. DUX25 total score and subscale scores

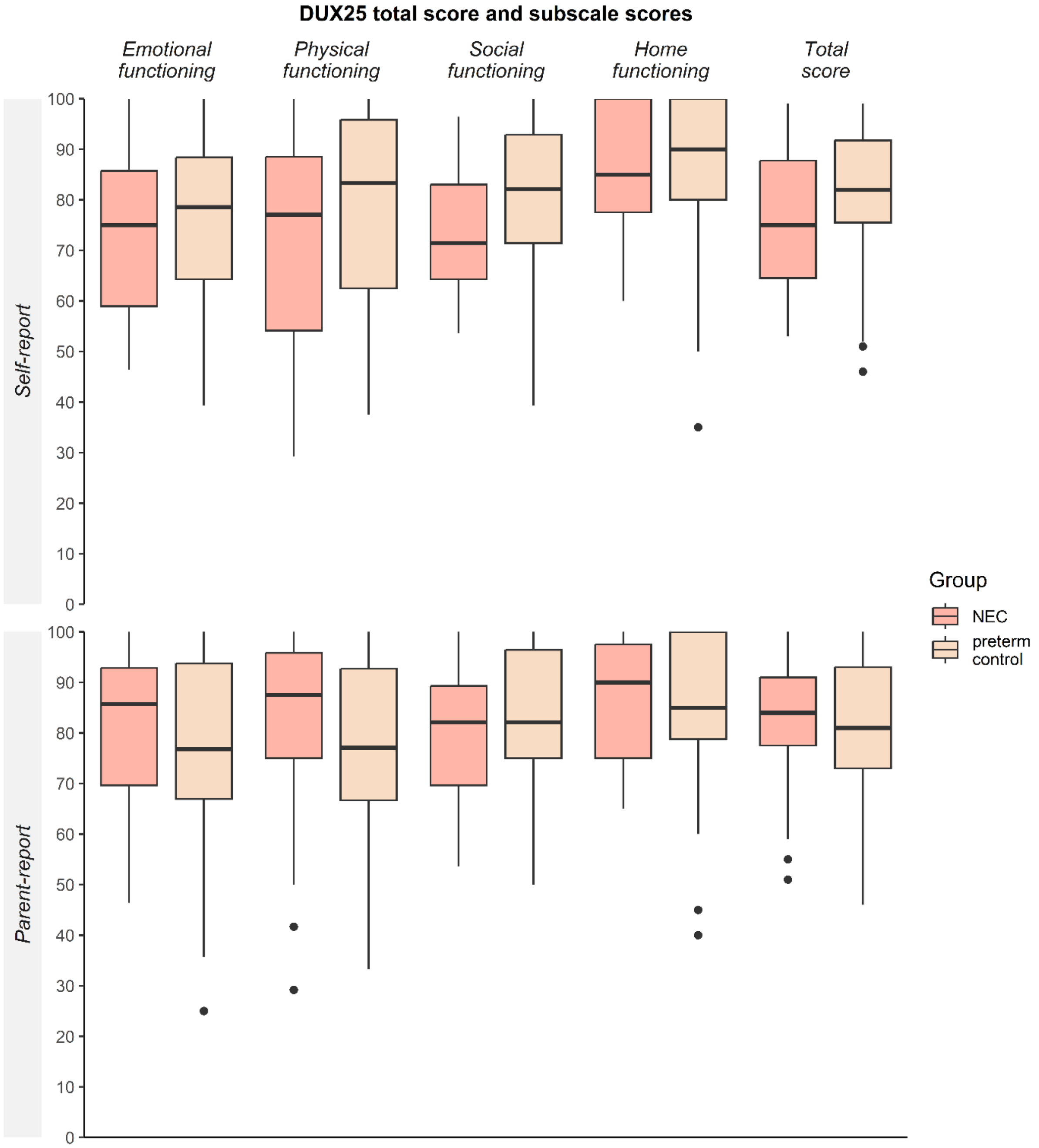

Supplementary figure S9. Correlation between self- and parent-reported DUX25 scores

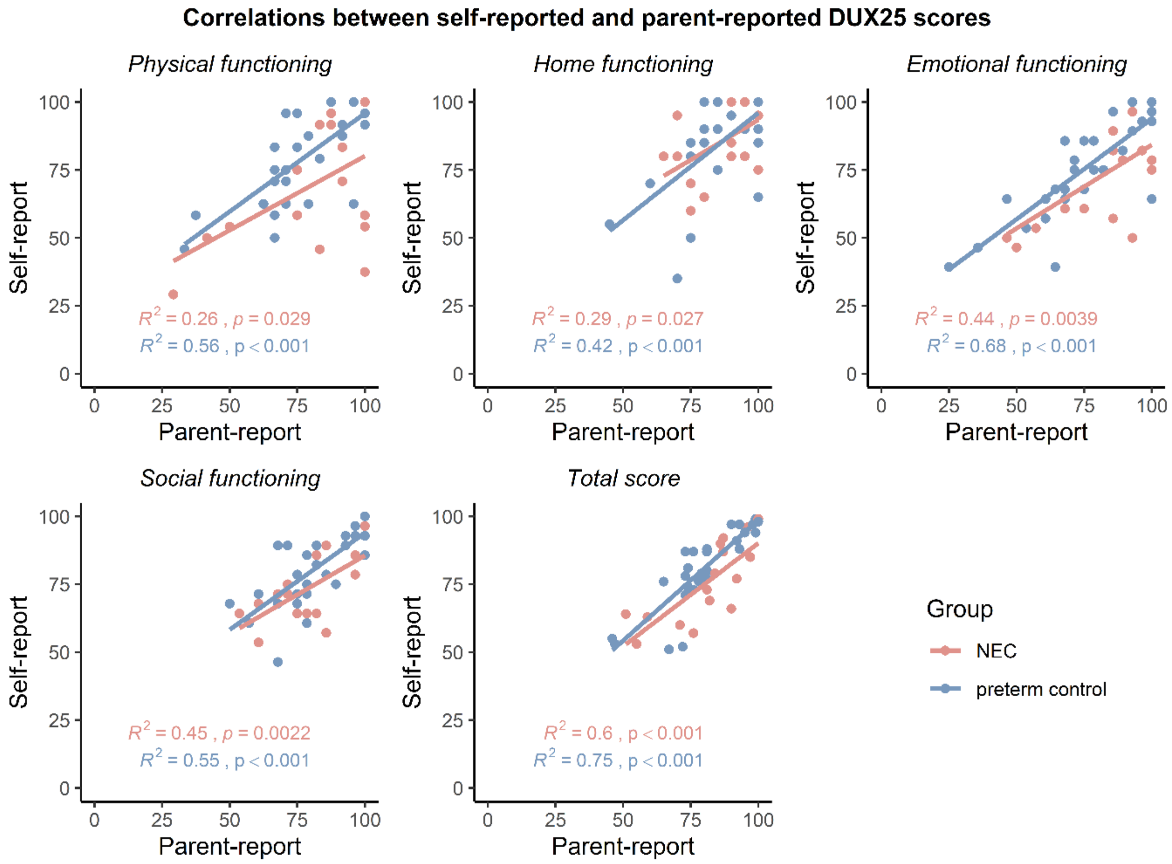

Supplement: Supplementary file 1 — Supplementary material [file 41390_2025_4348_MOESM1_ESM.pdf]
